# Postoperative simple biochemical markers for prediction of bone metastases in Egyptian breast cancer patients

**DOI:** 10.3332/ecancer.2013.305

**Published:** 2013-04-15

**Authors:** Nadia YS Morcos, Nadia I Zakhary, Mahmoud M Said, May MM Tadros

**Affiliations:** 1 Biochemistry Department, Faculty of Science, Ain Shams University, Egypt; 2 Cancer Biology Department, National Cancer Institute, Cairo University, Egypt

**Keywords:** Breast cancer, bone metastasis, inflammation markers, vascularisation markers

## Abstract

**Objective:**

The present study was undertaken to identify patient populations at high risk for bone metastases (BM) at any time after diagnosis of operable breast cancer.

**Subjects and methods:**

A total number of 59 cases with breast cancer after mastectomy was subdivided into two main groups that included 30 patients with radiologically confirmed BM and 29 patients with no bone metastasis (NBM). Patients with NBM were formerly observed for a one-year follow-up interval to monitor the development of bone metastasis (new BM). Parameters included a full blood picture, tumour markers (carcinoembryonic antigen and CA 15.3) and some biochemical markers (vascular endothelial growth factor and zinc levels, as well as tartrate-resistant acid phosphatase and alkaline phosphatase activities).

**Results:**

A significant elevation was recorded in carcinoembryonic antigen level and alkaline phosphatase activity, as well as inflammation and vascularisation markers at the time of primary diagnosis in patients with BM, compared with those without BM. CA 15.3 was significantly higher in the new BM group as compared with the other two groups (patients free of bone metastasis [free BM] and BM). According to the likelihood ratio, a panel of single, calculated as well as combined markers was proposed to predict BM within one year in breast cancer patients.

**Conclusion:**

Vascularisation and inflammation markers, as well as CA 15.3 are predictive of bone recurrence within one year in breast carcinoma patients. We suggest that in cancer validation studies it is imperative to search for markers that link to the premetastatic process and to determine what type of mechanism is active in each stage.

## Introduction

Breast cancer in women is the most common type of cancer-related mortality among Egyptian women worldwide [1]. According to GLOBOCAN 2008, breast cancer accounts for 38% of all new cancer cases in Egypt [2]. The median age at diagnosis is one decade younger than in Europe and North America and a good number of patients are premenopausal [3]. Sadly, the lack of public awareness and the financial shortages that impede screening and diagnostic services make the chances of survival lower and mortality rates correspondingly high [4]. 

The process of breast cancer metastasis, including tumour cell seeding, tumour dormancy, and metastatic growth, is only partly understood [5]. Bone is the first site of distant disease in 25–40% of patients with metastatic breast cancer, and up to 60–80% of patients with recurrent breast cancer eventually show evidence of skeletal involvement [6]. 

Complex biological pathways, including inflammation [7, 8], angiogenesis [9], invasion [10], osteoclastic activation, and bone matrix degradation, are involved in the formation of bone metastases [5]. Although the current diagnosis of bone metastases relies on bone imaging techniques, they are not sensitive enough for early detection and are invasive and expensive to use [11]. 

A flurry of surrogate biomarkers to detect micrometastasis has been developed in the last decade. These biomarkers open avenues for understanding cancer dormancy and metastasis, and may help predict outcome and therapeutic decisions at diagnosis and during follow-up of cancer patients [12]. 

The aim of the present study is to investigate prospectively whether simple markers and their combination could be used as a tool to detect and predict bone metastasis in breast carcinoma patients. 

## Patients and methods

### Patients

Egyptian women aged 30–69 years were recruited for this study (2007–2011) and selected from the outpatient clinic at the Clinical Pathology Department of the National Cancer Institute (NCI), Cairo University, Egypt. Physical examinations were processed by physicians in the Medical Oncology Unit, and routine clinical and pathological examinations were done. Once they were fully diagnosed and clinical staging of the disease was done according to the TNM classification of the American Joint Committee on Cancer (AJCC) TNM system, 59 patients were mastectomised and then scheduled for the appropriate treatment protocol of chemotherapy and radiotherapy. Of these patients, 29 had NBM and 30 had radiologically confirmed BM. Chemotherapy regimens included either FAC (5-fluorouracil + adriamycin + cyclophosphamide) or FEC (5-fluorouracil + epirubicin + cyclophosphamide) intravenously every 3 weeks for six cycles, and calcium supplements. Patients with BM received bisphosphonates, vitamin D, and calcium. Other therapeutic modalities included tamoxifen for hormone responsive patients, and Herceptin for human epidermal growth factor receptor-2 (HER2)-positive patients. Patients with NBM were observed for a one-year follow-up interval by bone scan to monitor the development of BM. All subjects gave written informed consent to participate in the study, which was carried out in accordance with the Helsinki Declaration. 

### Laboratory evaluations

Serum alkaline phosphatase (ALP) was determined by a kinetic method using an assay kit provided by Globe Diagnostic (Italy), plasma tartrate-resistant acid phosphatase (TRAP5b) activity by an immunoassay method using an ELISA kit (Immunodiagnostic Systems, UK) and plasma carcinoembryonic antigen (CEA) level by an electrochemiluminescence immunoassay ‘ECLIA’ method using an assay kit provided from Roche Diagnostics, USA. Serum vascular endothelial growth factor (VEGF) and CA 15.3 levels were determined by the double-antibody enzyme immunoassay methods using ELISA kits (Signosis, USA). Serum zinc concentration was evaluated by atomic absorption spectrophotometry using a 3100 Perkin Elmer Atomic Absorption Spectrometer (USA) in the Central Laboratory, Faculty of Science, Ain Shams University. 

A combination of previously reported markers was used in the present study that included monocytes% (monocyte count [10^7^/L]/WBC count [10^9^/L]) × 100 [13] and P2ms [(platelet count [10^9^/L])^2^/(monocyte fraction [%] × segmented neutrophil fraction [%])] [14]. Similarly, two new markers were proposed that include ALP/mono% (alkaline phosphatase/monocytes%) and VEGF/mono% (vascular endothelial growth factor/monocytes%). 

### Statistical methods 

The results were computed and statistics analysed using SPSS statistical software version 17.0 (SPSS Inc., Chicago, IL, USA). The quantitative results were expressed as median with minimum–maximum values. The non-parametric *Mann–Whitney U test *was used for comparing results between two groups (NBM versus BM), and the *Kruskal Wallis test *was used for comparing the quantitative results among free BM, new BM and BM groups. *Receiver operating characteristic (ROC) *curves were constructed, and the area under the curve (AUC) was calculated to assess the diagnostic accuracy of the markers. Cross-tabulation analysis was carried out and the significance (χ^2^) and likelihood ratio (LR) were calculated from the chosen cutoff values. The ROC analysis was first conducted on individual markers and then in combination to explore the potential that a panel of markers can provide improved performance [15]. 

## Results

Characteristics of patients are depicted in Table 1. There were no significant correlations between serum biomarkers and patient characteristics. Two breast cancer patients out of 30 from the BM group had visceral metastasis, which did not affect the overall result of the study. Markers chosen for the present study were selected to cover cancer dormancy and dissemination (CA 15.3 and CEA), angiogenesis and inflammation (VEGF, platelets, monocytes%, neutrophils%, zinc and ALP) and bone markers (TRAP5b and ALP). The results revealed that the four patients who developed bone metastasis within one year (new BM), although initially categorised with the NBM group, had a biochemical profile closer to those with radiologically confirmed bone metastasis (BM). Consequently, the results were compared once between the initial two groups (NBM versus BM), then recalculated by excluding the data of the four new BM patients from the NBM group. As a result, an assortment of data was attained, justifying the importance of choosing the appropriate conditions and samples for accurate validation. 

Analyses showed significantly elevated CEA level and ALP activity, as well as inflammation (ALP/mono% and P2ms), and vascularisation (VEGF/mono%) markers, and in contrast a significant decrease in monocytes% at the time of primary diagnosis in patients with BM, compared with those with NBM (Table 2).The CA 15.3 was significantly higher in the new BM group as compared with the other two groups (free BM and BM; p < 0.01) (Table 3). A cutoff value of 106 U/ml for CA 15.3 significantly differentiated between patients that newly developed BM within a one-year follow-up interval (100% of new BM patients had CA 15.3 values ≥ 106 U/ml) and those without bone involvement or initially diagnosed with BM (100% and 90% of free BM and BM patients had CA 15.3 < 106 U/ml, respectively) (χ^2^<0.001) (Table 4). No significant differences in TRAP5b, VEGF and zinc levels were recorded in all comparisons.

By using the cutoff value of 100 pg/ml for VEGF, it was possible to sort out 88.5% of free BM patients (<100 pg/ml), from 50% of patients who newly developed BM (≥100 pg/ml) (χ^2^<0.05) (Table 4). Consequently, we combined the VEGF and monocytes% as representative biomarkers for tumour angiogenesis. From ROC analysis, a ratio between VEGF and monocytes% (VEGF/mono%) was considered as an angiogenic marker with a cutoff value of 3.26. Cross-tabulation revealed that all patients who newly developed BM and those with initial BM had VEGF/mono% values ≥3.26, compared with only 26.7% of patients who remained free of BM (χ^2^<0.002) (Table 4). 

From the present study, we found that total ALP was elevated in all patients with bone metastasis; however, a significant increase was recorded in the BM group only, as compared with the free BM group (Table 3). A cutoff value of 119.2 U/L for serum ALP significantly differentiated between patients with BM and those without bone involvement (NBM; χ^2 ^<0.001) at the initial diagnosis. Accordingly, by calculating the ratio between ALP and monocytes%, and using a cutoff value of 4.9 for such ratio, we were able to differentiate between 72.7% of patients who were initially diagnosed with NBM (<4.9) and 90% of patients who had BM (≥4.9) (χ^2^<0.001). However, such differences did not reach significance when we compared free BM with new BM groups, probably due to the small number of patients in the latter group. Interestingly, by using the cutoff values of ALP + P2ms, we found that all patients without bone metastasis (free BM) had one or both markers below cutoff levels, whereas 66.7% of patients with new BM or 41.7% of all patients with bone metastasis (all BM) had both markers above the specified cutoff values. On the other hand, all patients with BM had both VEGF + ALP/mono% above cutoff levels, while 78.6% of free BM patients had one or both of the aforementioned parameters below the specified cutoff levels (χ^2 ^<0.001). In addition, cross-tabulation revealed that 95.8% of all BM patients had one or both of VEGF+ALP above cutoff levels, and in contrast 72% of free BM patients had both indices below cutoff levels (χ^2^ < 0.01) (Tables 4 and 5). 

The result of the current study revealed that the strength of markers (according to the likelihood ratio) in predicting bone metastasis within one year is in the following descending order: VEGF + ALP; VEGF + ALP/mono%; VEGF/mono%; monocytes%; CA 15.3; ALP + P2ms; P2ms and VEGF (Table 4). 

## Discussion

Metastasis is the leading cause of cancer death, and the metastatic cascade is a complex, yet inefficient process that we have only begun to understand recently [16]. Recent studies delineate a key role for the tumour microenvironment [17, 18] and cancer dormancy in cancer progression, and in consequence how to diagnose and treat the disease [19]. 

Many prognostic biomarkers discovered to date are usually evaluated as single markers, which lack sufficient specificity. We concur with Chechlinska’s argument that most cancer prognostic biomarker studies using modern technologies are methodologically flawed as they compare samples from cancer patients with those of healthy, inflammation-free people [20] or at best with those of patients with already metastasised tumours. In validation studies, it is important to determine whether the patient has dormant disease, and what type of mechanism is active, together with his or her systemic inflammatory response [21]. 

Comparing CA 15.3 and CEA tumour markers in NBM versus BM patients revealed that only CEA showed a significant (*p *< 0.05) increase in BM group; however, after redistributing the patients into three groups, we found that CA15.3 was significantly higher (*p *< 0.01) in the new BM group as compared with free BM and BM groups. This change in pattern was not observed for the CEA marker. Comparable outcomes were reported by Duffy [22] and Evangelista *et al *[23], who showed that an increase in CA 15.3 within one year is related to disease relapse. 

Our results concur with several studies, signifying that CA 15.3, which detects soluble forms of MUC1 oncoprotein, is valuable for monitoring therapy in patients with metastatic disease [24–27]. We also found that the rise in CA 15.3 was independent of tumour size, axillary nodal status and other sites of metastasis, which is in line with previous results [22, 28]. Contrary to Duffy *et al *[25], who revealed that the main limitation of CA 15.3 as a marker for breast cancer is that serum levels are rarely increased in patients with early or localised disease, we find that this actuality is suitable for identifying patients at risk to develop recurrence within one year. 

Inflammation and angiogenesis are hallmarks of cancer, and together they play important roles at all stages of cancer progression, where it is difficult to separate them from one another. Inflammation incites and promotes carcinogenesis by creating a tissue microenvironment that can enhance invasion, migration and angiogenesis [29]. The change in tumour phenotype from angiostatic to angiogenic is known as the angiogenic switch, which helps tumour to escape from dormancy. Under this concept, the balance of proangiogenic and antiangiogenic factors would ultimately determine the activation status of the switch [30]. Most solid tumours are infiltrated by different cells such as tumour-associated macrophages (TAMs), monocytes, neutrophils and platelets. It is speculated that the recruited monocytes differentiate into macrophages, which release numerous factors including VEGF that facilitates invasion [31, 32]. Similarly, neutrophils are recruited by tumour cells, where they secrete powerful proangiogenic factors including VEGF [33]. 

Different studies evaluated VEGF and monocytes percentage separately as vascularisation markers [20, 30, 34–36]. To the best of our knowledge, we proposed herein a ratio between VEGF and monocytes percentage as a new vascularisation marker for breast cancer. However, this ratio warrants further validation studies. 

Zinc (Zn) is an antioxidant, or free radical scavenger. Changes in zinc level in the blood of cancerous patients may be the cause of malignant tumour occurrence [37]. Another important role of zinc is in the process of angiogenesis, where it activates the matrix metalloproteinases (MMPs), especially under hypoxic conditions [38]. In the present study, although the blood zinc level was lower in patients with new BM, the differences among the studied groups did not reach significance. 

The contribution of platelets to the progression of cancer is an emerging area of research interest. Complex interactions between tumour cells and circulating platelets play an important role in cancer growth and dissemination, and a growing body of evidence supports a role for physiologic platelet receptors and platelet agonists in cancer metastases and angiogenesis [19, 39–41]. Boucharaba *et al *[42] characterised two roles of platelets in metastasis: as a direct source of tumour cell mitogens and as an indirect activator of osteoclastic activity in the bone microenvironment. 

The inverted correlation between platelets and monocytes% and segmented neutrophils% (known as P2ms) was recently used as a non-invasive test for hepatic fibrosis [14, 43, 44]. Correspondingly, we considered the possibility of using P2ms as a predictive marker of inflammation in breast cancer patients. To our knowledge, this is the first study validating the diagnostic accuracy of P2ms for detecting inflammation in breast cancer with BM. Cross-tabulation revealed that a cutoff value of 105.76 for P2ms significantly differentiated between patients who remained free of BM (88.9% of free BM patients had values <105.76) and those that newly developed or initially diagnosed with BM (66.7% of both patient groups had values ≥105.76). 

According to the data emerging from several studies, markers of bone metabolism may be an additional, useful, noninvasive diagnostic and prognostic tool to improve cancer disease management [45, 46]. Two different types of bone metabolism markers can be analysed: markers of bone resorption (such as tartrate-resistant acid phosphatase) and markers of bone formation (such as alkaline phosphatase). 

Tartrate-resistant acid phosphatase isoform 5b (TRAP5b) is a biochemical marker of osteoclast number and activity. Mounting evidence has demonstrated serum TRAP5b as a useful marker of bone resorption and is among one of the many markers that have been studied to be a surrogate marker of BM in cancer patients [45, 47, 48]. 

It was not possible to demonstrate a statistically significant difference in TRAP5b activity among all groups in the present study. Hung and Oremek [48] reported a similar conclusion. Other authors examined healthy women and breast cancer patients at primary diagnosis without signs of osseous metastases and did not find any difference in TRAP5b activity, whereas a significant difference was observed in patients in whom bone metastases were newly diagnosed [47, 49, 50]. These differences were explicated by Wu *et al *[51], who anticipated that serum TRAP5b activity may not be elevated in all breast cancer patients with BM, only in those with extensive metastasis. 

Total alkaline phosphatase (ALP) is a bone formation marker, used as a routine marker for skeletal involvement; however, different publications have pointed to the fact that bone-specific ALP is a better choice as an index of bone formation due to its higher specificity for bone [52]. Total ALP has been the most used marker for detecting increased bone formation in metastatic prostate and breast cancer [45, 46, 53]. ALP is also considered an inflammatory marker in cancer patients, and its elevation correlates with other inflammatory markers such as high C-reactive protein, low blood zinc levels, and reduction in overall survival of cancer patients [54–56]. In accordance with previous studies [54–56], our results show that inflammatory markers could be used for the prediction of bone recurrence in breast cancer patients. 

## Conclusion

Our relatively small study population limits the predictive power of the panels presented here, but the benefits of a serum biomarker and multimarker approach are clearly illustrated, and further studies utilising larger clinical cohorts are well warranted. This study demonstrates that a simple blood sample harbours information of tumour relapse in breast cancer patients and brings benefit to existing clinical predictors. We prove the importance of determining whether the patient has dormant disease, together with her systemic inflammatory response in order to improve the predictive marker validation. 

## Acknowledgments

The authors would like to thank Dr. Ola Mohamed Reda Khorshid, Assistant Professor of Medical Oncology, National Cancer Institute, Cairo University, for her cooperation and kind help in samples’ collection. 

## Figures and Tables

**Table 1:** Baseline characteristics of breast cancer patients included in the study.

Clinical Features		NBM (*n*=29)	BM (*n*=30)	χ^2^
**Age**	**(Mean±SD)**	**48.6 ± 9**	**52.2 ± 11**	**NS**
**Grade**	**1**	**1**	**1**	**NS**
	**2**	**22**	**19**	
	**3**	**4**	**7**	
	**4**	**2**	**3**	
**Stage**	**II**	**15**	**0**	**0.001**
	**III**	**13**	**2**	
	**IV**	**0**	**28**	
	**NA**	**1**	**0**	
**Estrogen receptor (ER)**	**(−)ve**	**7**	**7**	**NS**
	**(+)ve**	**22**	**23**	
**Progesterone receptor (PR)**	**(−) ve**	**4**	**12**	**0.05**
	**(+)ve**	**25**	**18**	
**Human epidermal growth factor receptor 2 (HER2)**	**(−)ve**	**6**	**3**	**0.05**
	**(+)ve**	**4**	**0**	
	**NA**	**19**	**27**	
**Lymph node**	**(−)ve**	**4**	**6**	**NS**
	**(+)ve**	**23**	**21**	
	**NA**	**2**	**3**	
**Visceral metastasis**	**NO**	**29**	**28**	**0.01**
	**YES**	**0**	**2**	
**Duration (years)**	**≥1 year**	**14**	**7**	**0.05**
	**2–4 years**	**10**	**14**	
	**≥5 years**	**5**	**9**	

NA, Not available; χ^2^, chi-square.

**Table 2:** Level of biochemical markers at the time of diagnosis in breast cancer patients.

		NBM (*n*=29)	BM (*n*=30)
**CA 15.3 (U/ml)**	**Median min–max**	**23.9 8.4–209**	**23.2 2.3- >250**
**CEA (ng/ml)**	**Median min–max**	**1.75 0.50–69**	6.4* 1.4- >100
**ALP (U/L)**	**Median min–max**	**92.5 31–287**	146.5** 41- >400
**TRAP5b (U/L)**	**Median min–max**	**2.15 0.62–18.4**	**2.02 0.20–8.36**
**VEGF (pg/ml)**	**Median min–max**	**52.49 17.2–142.4**	**74.91 16.1- >200**
**Zinc (mg/L)**	**Median min–max**	**0.46 0.19–1.74**	**0.45 0.2–2.03**
**Monocytes%**	**Median min–max**	**28.70 0.96- >80**	9.3* 1.38-47.8
**P2ms**	**Median min–max**	**9.3 3–379**	128* 9.2- >400
**ALP/mono%**	**Median min–max**	**3.8 1.26–44.8**	11.4* 3.1- >60
**VEGF/mono%**	**Median min–max**	**1.9 0.47–77**	17.7 ** 5.4–50

* p<0.05.

** p<0.01, Mann–Whitney U test.

**Table 3:** Level of biochemical markers at the time of diagnosis of breast cancer patients who remained free, developed or had bone metastases within one year.

		Free BM (*n*=25)	New BM (*n*=4)	BM (*n*=30)
**CA 15.3 (U/ml)**	**Median min–max**	**22.2 8.4–102.1**	159.6 ^a^** 110–209.2	23.2 ^b^** 2.3- >250
**CEA (ng/ml)**	**Median min–max**	**1.5 0.50–69**	**6.4 1.2–16.8**	6.4 ^a^* 1.4- >100
**ALP (U/L)**	**Median min–max**	**86.0 31–287**	**129.5 63–218**	146.5 ^a^** 41- >400
**TRAP5^b^ (U/L)**	**Median min–max**	**2.15 0.92–18.4**	**2.10 0.60–3.24**	**2.02 0.20–8.36**
**VEGF (pg/ml)**	**Median min–max**	**52.49 17.2–142.4**	**80.90 29. 5–127.2**	**74.91 16.1 ->200**
**Zinc (mg/L)**	**Median min–max**	**0.46 0.19–1.74**	**0.38 0.28–0.47**	**0.45 0.2–2.03**
**Monocytes%**	**Median min–max**	**28.70 0.96- >80**	**10.7 6.25–34.2**	9.3 ^a^** 1.38–47.8
**P2ms**	**Median min–max**	**9.3 3–379**	**34.3 4.8–233**	128 ^a^* 9.2- >400
**ALP/mono%**	**Median min–max**	**3.8 1.26–44.8**	**20.4 18.4–25**	11.4 ^a^** 3.1- >60
**VEGF/mono%**	**Median min–max**	**1.9 0.47–77**	**4.7 3.4–11.9**	17.7 ^a^** 5.4–50

a Results versus free BM.

b Results versus new BM.

* *p* < 0.05.

** *p* < 0.01, Kruskal Wallis test.

**Table 4:** Cross-tabulation showing the reliability of significant markers (single, calculated and combined) in predicting bone metastasis within one year among all groups.

Marker	Cutoff	Free BM (%)	New BM (%)	BM (%)	χ^2^	LR
**CA 15.3 (U/ml)**	**<106**	**100**	**0**	**90**	**0.001**	**12**
	**≥106**	**0**	**100**	**10**		
**Monocytes%**	**>12.3**	**78.9**	**33.3**	**10**	**0.001**	**14.4**
	**≥12.3**	**21.1**	**66.7**	**90**		
**P2ms**	**<105.76**	**88.9**	**33.3**	**33.3**	**0.01**	**10.4**
	**≥105.76**	**11.1**	**66.7**	**66.7**		
**VEGF (pg/ml)**	**<100**	**88.5**	**50**	**62.5**	**0.05**	**5.9**
	**≥100**	**11.5**	**50**	**37.5**		
**VEGF/mono%**	**<3.26**	**73.3**	**0**	**0**	**0.01**	**15.7**
	**≥3.26**	**26.7**	**100**	**100**		
**VEGF + ALP**	**Both low**	**72**	**25**	**0**	**0.001**	**31.3**
	**One or both high**	**28**	**75**	**100**		
**ALP + P2ms**	**One or both low**	**100**	**33.3**	**66.7**	**0.01**	**11.4**
	**Both high**	**0**	**66.7**	**33.3**		
**VEGF + ALP/mono%**	**One or both low**	**78.6**	**0**	**0**	**0.001**	**17.3**
	**Both high**	**21.4**	**100**	**100**		

LR, likelihood ratio; χ^2^, chi-square.

**Table 5:** Cross-tabulation showing the reliability of combined markers in predicting bone metastasis within one year (free BM versus all BM [BM + new BM]).

Marker	Cutoff	Free BM (%)	All BM (%)	χ^2^	LR
**VEGF + ALP**	**Both low**	**72**	**4.2**	**0.01**	**27.5**
	**One or both high**	**28**	**95.8**		
**ALP + P2ms**	**One or both low**	**100**	**58.3**	**0.01**	**10.4**
	**Both high**	**0**	**41.7**		
**VEGF + ALP/mono%**	**One or both low**	**78.6**	**0**	**0.001**	**17.3**
	**Both high**	**21.4**	**100**		
